# Neugeborenen-Hörscreening in Deutschland – Ergebnisse der Evaluationen 2011/2012 und 2017/2018

**DOI:** 10.1007/s00103-023-03779-0

**Published:** 2023-10-16

**Authors:** Inken Brockow, Kristina Söhl, Marianne Hanauer, Annette Heißenhuber, Carola Marzi, Antoinette am Zehnhoff-Dinnesen, Peter Matulat, Ulrich Mansmann, Uta Nennstiel

**Affiliations:** 1 GP1, Bayerisches Landesamt für Gesundheit und Lebensmittelsicherheit (LGL), Veterinärstr. 2, 85764 München-Oberschleißheim, Deutschland; 2 Klinik für Phoniatrie und Pädaudiologie, Westfälische Wilhelms-Universität Münster (UKM), Münster, Deutschland; 3 Institut für Informationsverarbeitung, Biometrie und Epidemiologie (IBE), Ludwig-Maximilians-Universität (LMU), München, Deutschland

**Keywords:** Neugeborene, Hörscreening, Tracking, Lost to Follow-up, Evaluation, Hörstörungen, Qualitätssicherung, Screening, Newborn, Hearing screening, Lost to follow-up, Evaluation, Quality criteria, Hearing disorder, Quality assurance, Screening

## Abstract

**Hintergrund:**

Das Neugeborenen-Hörscreening (NHS) wurde 2009 durch den Gemeinsamen Bundesausschuss (G-BA) mit Aufnahme in die Kinder-Richtlinie bundesweit eingeführt. Dabei wurden in der Kinder-Richtlinie auch Qualitätsziele festgelegt. Um die Qualität des NHS in Deutschland zu überprüfen, hat der G‑BA eine Bietergemeinschaft mit einer ersten Evaluation für die Jahre 2011/2012 und einer Folge-Evaluation für 2017/2018 beauftragt.

**Methoden:**

Grundlage der Evaluationen waren Sammelstatistiken, die von allen geburtshilflichen und neonatologischen Abteilungen, als Leistungserbringer des NHS, geführt werden müssen und ggf. in Kooperation mit Hörscreening-Zentralen (HSZ) erstellt werden. Zusätzliche Daten wurden durch Fragebögen und Interviews erhoben und durch Routinedaten ergänzt, um den vollständigen Screeningprozess zu evaluieren.

**Ergebnisse:**

In 13 Bundesländern sind insgesamt 15 HSZ in den Screeningprozess eingebunden. Deutschlandweit wurde 2018 eine Screeningrate von 86,1 % (2012: 82,4 %) dokumentiert, die sich deutlich zwischen den Bundesländern unterschied. Die vorgegebenen Qualitätsziele konnten noch nicht überall umgesetzt werden. So erreichten nur knapp die Hälfte der Geburtsabteilungen die angestrebte Screeningrate von über 95 %. Beim Vergleich der Folge-Evaluation mit den Daten der ersten Evaluation konnte gezeigt werden, dass sich die Strukturqualität des NHS verbessert hatte, während die Prozessqualität eher gleich blieb oder schlechter geworden war, verdeutlicht insbesondere durch einen Anstieg der Refer-Rate (Kinder, die mit einem auffälligen Befund entlassen wurden) von 5,3 % auf 6,0 %.

**Diskussion:**

Zur Verbesserung der Qualität des NHS sollten flächendeckend HSZ etabliert und – wie in der Richtlinie vorgesehen – bei auffälligem Erstscreening ein zweites Screening noch vor Entlassung konsequenter durchgeführt werden.

## Abstract (en)

**Background:**

Newborn hearing screening (NHS) was introduced nationwide by the Federal Joint Committee (Gemeinsamer Bundesausschuss, G‑BA) in 2009. In this process, quality targets were also set in the pediatrics directive. In order to review the quality NHS in Germany, the G‑BA commissioned a consortium to conduct an initial evaluation for the years 2011 and 2012 and a follow-up evaluation for 2017 and 2018.

**Methods:**

The evaluations were based on NHS screening parameters (Sammelstatistiken) that must be documented by all obstetrics and neonatology departments as NHS providers and can also be compiled through cooperation with hearing screening centers (HSCs). Additional data were collected through questionnaires and interviews and routine data were used to evaluate the screening process.

**Results:**

In 13 federal states, a total of 15 HSCs are involved in the screening process. Across Germany, an NHS screening rate of 86.1% was documented in 2018 (82.4% in 2012), but this differed significantly between the federal states. The specified quality targets could not yet be implemented everywhere. For example, only less than half of the obstetric departments achieved the specified screening rate of over 95%. A comparison of data from the follow-up evaluation and the first evaluation showed that the structural quality of NHS had improved, while the process quality remained the same or had deteriorated. The refer rate (children who were discharged without passing the screening) increased from 5.3% to 6.0%.

**Discussion:**

To improve the quality of NHS, HSCs should be established nationwide and a second screening should be carried out more consistently before discharge in the case of a refer result in the initial screening.

## Hintergrund

In Deutschland sind 1 bis 2 von 1000 Neugeborenen von einer behandlungsbedürftigen beidseitigen Hörstörung betroffen [1]. Für eine altersgerechte lautsprachliche Entwicklung dieser Kinder sind die frühzeitige Diagnose und Therapie der Hörstörung entscheidend. Wird die Diagnose verspätet gestellt, zeigen sich bei den betroffenen Kindern z. B. auch Nachteile in der grobmotorischen und sozialen Entwicklung, eine geringere Lebensqualität und eine noch im Teenageralter nachweisbare schlechtere Lesekompetenz [2–6]. Mit der Messung transitorisch evozierter otoakustischer Emissionen (TEOAE) oder einer Hirnstammaudiometrie („automated auditory brainstem response“, AABR) stehen automatisierte Verfahren für ein Hörscreening in den ersten Lebenstagen zur Verfügung [7]. Vor diesem Hintergrund hat der Gemeinsame Bundesausschuss (G-BA) 2009 bundesweit ein Neugeborenen-Hörscreening (NHS) in die Kinder-Richtlinie und damit in die Regelversorgung aufgenommen [8]. In der Kinder-Richtlinie wurden neben Vorgaben für die Durchführung des NHS auch Qualitätsziele festgelegt. So sollen mehr als 95 % der Neugeborenen gescreent und 95 % der Kinder mit einem auffälligen Erstscreeningbefund noch vor Entlassung aus der Klinik ein Kontrollscreening mit einer AABR erhalten sowie höchstens 4 % der Kinder mit einem auffälligen Befund entlassen werden (Refer-Rate). Die Diagnose einer beidseitigen Hörstörung mit einem Hörverlust über 35 dB soll bis zum 3. Lebensmonat gestellt und die Therapie bis zum 6. Lebensmonat eingeleitet werden.

Alle Leistungserbringer des NHS (in erster Linie geburtshilfliche und neonatologische Abteilungen) müssen nach der Kinder-Richtlinie sogenannte Sammelstatistiken mit definierten Screeningparametern des NHS erstellen. In 13 Bundesländern sind insgesamt 15 Hörscreening-Zentralen (HSZ) in den Prozess eingebunden. Diese übernehmen für die angebundenen Kliniken die Dokumentation der Screeningparameter und weitere Aufgaben der Qualitätssicherung. Dazu gehören in den meisten Zentralen die Sicherstellung der Vollständigkeit des NHS und die Erinnerung der Eltern an notwendige Kontrolluntersuchungen nach einem auffälligen Screeningbefund (Tracking).

Um die Qualität der Umsetzung des NHS zu untersuchen, hatte der G‑BA 2014 eine Bietergemeinschaft unter Federführung des Bayerischen Landesamts für Gesundheit und Lebensmittelsicherheit (LGL) mit einer bundesweiten Evaluation des NHS für die Jahre 2011 und 2012 beauftragt. Bei dieser ersten Evaluation sollte der gesamte Screeningprozess in Hinblick auf Struktur‑, Prozess- und Ergebnisqualität bewertet werden. Die Ergebnisse hatten gezeigt, dass das Hörscreening insgesamt gut umgesetzt wurde, aber einige der Qualitätsanforderungen noch nicht erreicht waren. Ende 2019 wurde daher vom G‑BA eine Folge-Evaluation der Jahre 2017/2018 an dieselbe Bietergemeinschaft in Auftrag gegeben. Im Rahmen der Folge-Evaluation sollte die Prozessqualität des NHS erneut evaluiert und zugleich geprüft werden, ob sich diese seit der ersten Evaluation verbessert hat.

Ziel dieser Arbeit ist es, die Ergebnisse der beiden Evaluationen darzustellen und die sich daraus ergebenden Herausforderungen für die Umsetzung und weitere Qualitätsverbesserung des NHS in Deutschland zu beschreiben.

## Methoden

Sowohl für die erste Evaluation 2011/2012 als auch für die Folge-Evaluation 2017/2018 wurden über mehrere Datenquellen die geburtshilflichen und neonatologischen Abteilungen identifiziert [9]. Anschließend wurden von diesen Leistungserbringern mithilfe eines Fragebogens (in der ersten Evaluation als PDF, in der Folge-Evaluation über einen mit LimeSurvey erstellten Onlinefragebogen) Angaben zu vorhandenen Screeninggeräten und zur Organisation des NHS erhoben. Von Abteilungen, die nicht an eine HSZ angebunden waren, wurden über die Fragebögen auch die Sammelstatistiken standardisiert angefordert. Für die angebundenen Abteilungen stellten 13 der 15 HSZ statt der Sammelstatistiken anonymisierte Einzeldatensätze für den gesamten Screeningprozess eines Kindes bis zur endgültigen Diagnose nach einer vorgegebenen Variablenliste bereit, die anderen beiden HSZ übermittelten nur kumulative Daten, ähnlich den Sammelstatistiken. Die HSZ wurden auch zu Aufgaben, Organisation und Finanzierung befragt. Da in der ersten Evaluation der gesamte Screeningprozess evaluiert werden sollte, wurden in 9 pädaudiologischen Institutionen Eltern zu ihren Erfahrungen mit dem NHS und der Dokumentation im gelben Kinderuntersuchungsheft befragt. Außerdem wurden alle pädaudiologischen Institutionen um anonymisierte Daten der Kinder gebeten, bei denen eine beidseitige Hörstörung in ihrer Institution diagnostiziert worden war. Diese beiden Befragungen entfielen in der Folge-Evaluation, stattdessen wurden mit 10 Vertreterinnen und Vertretern aus geburtshilflichen und neonatologischen Abteilungen qualitative Interviews geführt. Weiterhin wurden Sekundärdaten genutzt, wie z. B. Daten der Bevölkerungsstatistik des Statistischen Bundesamtes, Daten aus dem Qualitätsbericht der außerklinischen Geburtshilfe in Deutschland (QUAG) und Daten des Zentralinstituts für die kassenärztliche Versorgung in der Bundesrepublik Deutschland (Zi) zu ambulant abgerechneten NHS-Untersuchungen.

Für die Berechnung der Screeningrate musste als Nenner die Zahl der Lebendgeborenen einer Klinik und nicht der Lebendgeborenen aus der Bevölkerungsstatistik nach Wohnort herangezogen werden, da auch die Hörscreeningdaten auf Klinik- oder Abteilungsebene dokumentiert werden. In der ersten Evaluation wurde die Zahl der Lebendgeborenen aus den Daten der externen stationären Qualitätssicherung (esQS) zusammengestellt. In der Folge-Evaluation wurden die Geburtenzahlen einer Klinik der Nutricia-Milupa-Geburtenliste entnommen [10], da auf die entsprechende Dokumentation beim Institut für Qualität und Transparenz im Gesundheitswesen (IQTIG) nicht zugegriffen werden konnte. Wann immer möglich, wurden für eine bessere Vergleichbarkeit in beiden Evaluationen die gleichen Definitionen und Berechnungen verwendet [11, 12]. Die Daten wurden mit IBM SPSS Version 23 bzw. 25 (IBM Corp., Armonk, NY, USA) aufbereitet und ausgewertet.

## Ergebnisse

Wenn nicht anders angegeben, werden im Vergleich zu den Ergebnissen von 2018 die Ergebnisse von 2012 in Klammern dargestellt.

### Datengrundlage

Für die Evaluationen wurden Hörscreeningdaten für insgesamt 1047 (1150) geburtshilfliche und neonatologische Abteilungen von den Leistungserbringern bzw. Hörscreening-Zentralen angefordert, für 86,0 % (82,4 %) der Abteilungen lagen Angaben aus den Fragebögen vor. Zwischen der ersten Evaluation und der Folge-Evaluation wurden 97 geburtshilfliche und 6 neonatologische Abteilungen geschlossen.

**Figure 1:**
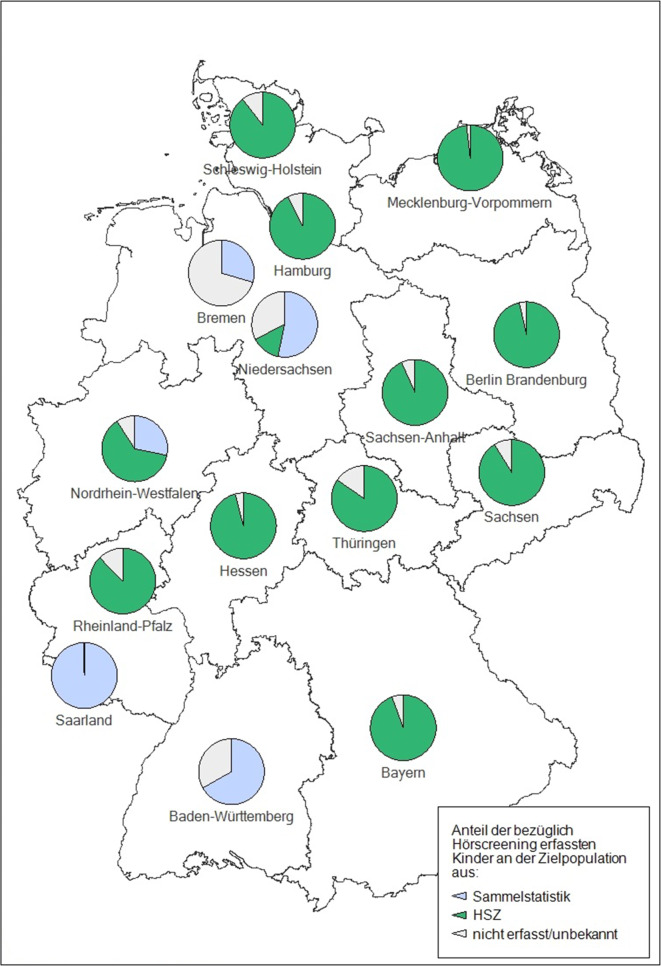

70,3 % (66,5 %) der Abteilungen arbeiteten mit einer Hörscreening-Zentrale (HSZ) zusammen. Somit konnten 2018 die Daten von etwa 240.000 Lebendgeborenen (etwa 30 %) in Deutschland nicht über eine HSZ erfasst werden. In 11 Bundesländern kooperierten nahezu alle Kliniken mit einer HSZ, in 2 Bundesländern (Nordrhein-Westfalen und Niedersachsen) nur ein Teil der Kliniken. In 2 Bundesländern (Bremen, Saarland) gab es noch nie eine HSZ (Abb. 1). Die Zentrale in Hamburg hat zum 01.01.2019 wegen Personalmangels und unklarer Weiterfinanzierung die Arbeit beendet. Zum selben Zeitpunkt hat eine Zentrale für Baden-Württemberg ihre Tätigkeit aufgenommen. Alle HSZ sind im Verband der Deutschen Hörscreening-Zentralen (VDHZ) organisiert.

### Vollständigkeit des NHS

Insgesamt wurde in Deutschland für 86,1 % (82,4 %) der Neugeborenen ein Hörscreening dokumentiert. Es ist nicht bekannt, ob bei den übrigen Neugeborenen das Hörscreening nur nicht dokumentiert („lost to documentation“) oder wirklich nicht durchgeführt wurde. Die dokumentierte Screeningrate unterschied sich zwischen den Bundesländern mit 29,5–99,7 % (52,9-98,9 %) erheblich und war in der Regel für Bundesländer mit nahezu flächendeckender Anbindung an eine HSZ deutlich höher (Abb. 1). Eine Screeningrate konnte für 83,6 % (69,3 %) aller Geburtsabteilungen berechnet werden; von diesen erreichten 42,4 % (44,0 %) die in der Richtlinie als Qualitätsziel geforderte Screeningrate von über 95 %. Als Gründe für eine hohe Screeningrate in einer Abteilung wurden in den Interviews der Folge-Evaluation ausreichendes und gut geschultes Personal, Flexibilität beim Zeitpunkt des NHS sowie einfach zu bedienende Messgeräte genannt. In 45,3 % (56,4 %) der Kliniken ohne Anbindung an eine HSZ war nicht bekannt, dass eine Sammelstatistik mit den Hörscreeningparametern zu führen ist. Die Daten der Sammelstatistiken waren häufiger unvollständig oder unplausibel.

### Refer-Rate

Nach der Kinder-Richtlinie soll bei einer Geburt im Krankenhaus der Anteil der Kinder, die mit einem kontrollbedürftigen Befund entlassen werden (Refer-Rate), bei höchstens 4 % liegen. Dies kann durch eine gute Messqualität und die konsequente Durchführung eines 2‑stufigen Screenings, bei dem auffällige Ergebnisse eines ersten Hörtests noch vor Entlassung aus der Klinik mit einem weiteren Hörtest (Rescreening) kontrolliert werden, erreicht werden. Die Refer-Rate lag bundesweit bei 6,0 % (5,3 %). Die in der Kinder-Richtlinie als Qualitätsziel festgelegte Refer-Rate von unter 4 % erreichten 44,1 % (49,4 %) der Geburtsabteilungen, für die eine Refer-Rate berechenbar war (2018: 88,2 %, 2012: 77,7 %).

In der Kinder-Richtlinie wird ein Rescreening vor Entlassung mit einer AABR bei 95 % aller Kinder mit einem auffälligen Erstscreening gefordert. Da in den Sammelstatistiken nur kumulative Daten erfasst werden und so eine Zuordnung von Erst- und Rescreening bei einem Kind nicht möglich ist, ergeben sich aus diesen Daten keine validen Rescreening-Raten (Tab. 1). Die Rescreening-Raten aus Daten der HSZ lagen bei 47,4 % (30,9 %), d. h., über 50 % der Kinder mit einem auffälligen Befund haben kein Rescreening erhalten. In 54,2 % (54,6 %) der als Rescreening dokumentierten Messungen wurde, entgegen der Vorgaben der Kinder-Richtlinie, als Methode eine TEOAE verwendet. In den Interviews wurde diese Abweichung mit fehlenden AABR-Geräten, organisatorischen Gründen und der schnelleren, einfacheren und weniger störanfälligen Messung mit TEOAE begründet. Bei einem Wechsel der Screeningmethode für das Rescreening war die Rate auffälliger Ergebnisse höher. Bei der Methodenfolge AABR-AABR lag sie bei 14,0 %, bei TEOAE-AABR bei 26,6 % und am niedrigsten mit 9,6 % bei TEOAE-TEOAE.

**Table 1:** 

Bundesland	Datenquelle	Lebendgeborene^c^	Dokumentierte Screenings	Auffälliges Erstscreening (Refer)	Rescreening		Auffälliges Rescreening		Auffälliges Endergebnis		Abschlussbefund nicht bekannt	
		*n*	*n*	*n*	*n*	%	*n*	%	*n*	%	*n*	%
Baden-Württemberg	Sammelstatistiken	108.989	72.798	5736	2809	–	886	31,5	3989	6,4	–	–
Bayern	HSZ	127.689	120.570	10.795	6455	59,8^d^	1368	21,2	5733	4,8	558	9,7
Berlin/Brandenburg	HSZ	59.519	57.411	5668	1009	17,8^d^	78	7,7	4742	8,3	4569	96,4
Bremen	Sammelstatistiken	9963	2936	336	12	–	k.A.	–	324	11,4	–	–
Hamburg	HSZ^a^	25.790	23.867	k. A.	k. A.	k. A.	k. A.	k. A.	673	2,8	673	100,0
Hessen	HSZ	58.758	56.384	10.617	9075	85,5	944	10,4	2606	4,6	555	21,3
Mecklenburg-Vorpommern	HSZ	13.031	12.796	671	325	48,4^d^	85	26,2	432	3,4	207	47,9
Niedersachsen	HSZ Oldenburg^a^	69.711	6392	k. A.	k. A.	k. A.	k. A.	k. A.	672	10,5	176	26,2
	HSZ Vechta^a^		3514	k. A.	k. A.	k. A.	k. A.	k. A.	319	9,1	319	100,0
	Sammelstatistiken		37.114	3499	2292	–	563	24,6	2182	6,2	–	–
Nordrhein-Westfalen	HSZ Nordrhein	172.484	49.429	16.111	15.572	96,6	3060	19,7	3923	7,9	1869	47,6
	HSZ Westfalen-Lippe		58.633	16.174	14.946	92,4	2712	18,2	4267	7,3	2601	61,0
	Sammelstatistiken		48.652	4573	3055	–	1059	34,7	3088	6,7	–	–
Rheinland-Pfalz	HSZ^a^	35.795	31.361	k. A.	k. A.	k. A.	k. A.	k. A.	2287	7,3	2287	100,0
Saarland	Sammelstatistiken	9243	9214	692	1418	–	136	9,6	520	6,0	–	–
Sachsen	HSZ Dresden	35.566	21.476	1208	334	27,6^d^	41	12,3	916	4,3	847	92,5
	HSZ Leipzig		10.936	691	263	38,1^d^	39	14,8	467	4,3	281	60,2
Sachsen-Anhalt	HSZ	17.954	16.745	1322	707	53,5^d^	80	11,3	770	4,6	318	41,3
Schleswig-Holstein	HSZ	21.520	19.257	2211	1696	76,7^d^	146	8,6	705	3,7	254	36,0
Thüringen	HSZ Thüringen^b^	16.980	14.364	812	82	10,1^d^	38	46,3	748	5,2	312	40,7
*Deutschland*	*Gesamt*	*782.992*	*673.849*	*81.116*	*60.050*	*47,4*^*d*^	*11.235*	*18,7*	*39.363*	*6,0*	*15.826*	*54,1*

*k.* *A.* keine Angabe – Angaben sind aus Sammelstatistiken nicht möglich

^a^Nur das Endergebnis des Screenings wird erfasst

^b^Eine Erfassung in Einzeldatensätzen in der Hörscreening-Zentrale (HSZ) erfolgt nur im Falle eines auffälligen Erstscreenings

^c^Lebendgeborene nach Geburtsort: [31, 32]

^d^Anteil für Hörscreening-Zentralen mit Einzeldatensätzen und validen Angaben des Rescreenings

Einige HSZ (Hessen, Nordrhein, Westfalen-Lippe) wurden bei der Berechnung der Rescreening-Rate nicht berücksichtigt, da sie beim Erstscreening jeden ersten übermittelten Testversuch als eigenes Testergebnis und den letzten Versuch einer Testreihe als Rescreening werteten. Dadurch ist bei diesen HSZ sowohl der Anteil der auffälligen Erstscreenings als auch die Rescreening-Rate sehr hoch (Tab. 1). Die Abgrenzung zwischen Erst- und Rescreening empfanden auch die Interviewten in den Kliniken als schwierig.

### Weitere Diagnostik

Nur in den HSZ kann die weitere Abklärung eines auffälligen NHS nach Entlassung aus der Klinik bei einem Kind dokumentiert werden. Eine detaillierte Erfassung aller Kontrolluntersuchungen bis zur endgültigen Diagnosestellung war nur Auftrag der ersten Evaluation. Hier zeigte sich, dass eine erste Kontrolluntersuchung nach auffälligem Screening häufig in einer HNO- oder pädiatrischen Praxis durchgeführt wurde und nicht – wie in der Richtlinie vorgesehen – durch eine pädaudiologische Institution oder pädaudiologisch qualifizierte HNO-Praxen. Das Ergebnis dieser ersten Kontrolluntersuchung war 2012 in 82,9 % unauffällig.

In beiden Evaluationen wurde der Abschlussbefund in den HSZ erfragt. Bei 2,4 % (3,7 %) der Kinder mit auffälligem Screening bei Entlassung wurde eine permanente Hörstörung diagnostiziert und bei 43,6 % (56,2 %) ausgeschlossen. Jedoch war deutschlandweit bei 54,1 % (40,1 %) kein abschließendes Ergebnis in den HSZ bekannt. Der Anteil der Kinder mit unklarer Abklärung des auffälligen Hörscreenings variierte sehr zwischen den HSZ. Während er in Bayern 2018 nur 9,7 % betrug, lag der Anteil in anderen Bundesländern bei bis zu 100,0 % (Tab. 1). In einigen HSZ ist grundsätzlich kein abschließender Befund bekannt, da das Tracking vorzeitig, z. B. mit der ersten Kontrolluntersuchung oder nach einem definierten Zeitraum, beendet wird.

Aus den in der ersten Evaluation angeforderten pädaudiologischen Daten konnte eine Prävalenz einer beidseitigen permanenten konnatalen Hörstörung von 1,3 pro 1000 Neugeborenen berechnet werden. Der Median des Diagnosealters lag 2011 bei 5 und 2012 bei 4 Monaten. Der in der Kinder-Richtlinie geforderte Therapiebeginn bis zum 6. Lebensmonat wurde 2011 bei 49,6 % und 2012 bei 54,2 % der Kinder erreicht.

### Vergleich der Ergebnisse der ersten Evaluation und der Folge-Evaluation

Eine zentrale Frage der Folge-Evaluation war, ob die in der Kinder-Richtlinie vorgegebenen Qualitätskriterien inzwischen erreicht wurden. Es konnte gezeigt werden, dass sich die Strukturqualität des Hörscreenings mit einer verbesserten Dokumentationsrate des NHS und vermehrter Anbindung der Abteilungen an eine Hörscreening-Zentrale positiv entwickelt hat. Dagegen hat sich die Qualität des Hörscreenings nicht verändert oder eher verschlechtert, was insbesondere der Anstieg der Refer-Rate von 5,3 % auf 6,0 % verdeutlicht (Tab. 2).

**Table 2:** 

	2012	2018
Qualitätsparameter	%	%
Rücklauf Fragebogen der Abteilungen	82,4	86,0
An Hörscreening-Zentrale (HSZ) angebundene Abteilungen	66,5	70,2
Notwendigkeit, Sammelstatistik zu führen, war in den Abteilungen ohne Anbindung an HSZ *nicht* bekannt	56,4	45,3
Dokumentierte Screeningrate auf Bundesebene (Teilnahmerate)	82,4	86,1
Screeningrate für geburtshilfliche Abteilung aus Sammelstatistik berechenbar	63,4	69,2
Anteil der geburtshilflichen Abteilungen mit einer Screeningrate über 95 %^a^	44,0	42,2
Anteil Erstscreening TEOAE	80,0	75,7
Anteil Erstscreening AABR	20,0	24,3
Anteil durchgeführtes Rescreening mit validen Angaben^b^	30,9	47,4
Anteil mit AABR durchgeführtes Rescreening	45,2	43,2
Refer-Rate auf Bundesebene	5,3	6,0
Anteil der geburtshilflichen Abteilungen mit einer Refer-Rate unter 4 %^a^	49,4	44,1

*TEOAE* transitorisch evozierte otoakustische Emissionen, *AABR* automated auditory brainstem response (Hirnstammaudiometrie)

^a^Bezogen auf Abteilungen mit berechenbarer Rate

^b^Ohne HSZ Hessen, Nordrhein und Westfalen; bei Berücksichtigung von weiteren Messungen in allen HSZ 2012: 74,8 %, 2018: 76,1 %

## Diskussion

Wie jedes populationsbasierte Screening muss das NHS nicht nur als Test, sondern als Programm mit kontinuierlichen Anpassungen und Verbesserungen verstanden werden [13–15]. Voraussetzung für die Qualitätssicherung ist eine valide Erfassung der Daten des gesamten Screeningprozesses [16]. Es ist davon auszugehen, dass tatsächlich mehr Kinder ein Hörscreening erhalten haben, als es nach der dokumentierten Screeningrate von 86 % erscheint. So war nur bei 1,86 % der Kinder dokumentiert, dass kein NHS durchgeführt wurde, während bei 12,3 % das NHS nicht erfasst wurde („lost to documentation“). Bei Durchführung des NHS im ambulanten Bereich, z. B. nach ambulanten Geburten, frühzeitiger Entlassung oder fehlendem Personal in der Klinik, erfolgt nur selten eine Übermittlung der Hörscreening-Daten an die Zentralen. Zudem war auch bei der Folge-Evaluation in fast der Hälfte der Abteilungen ohne Anbindung an eine Hörscreening-Zentrale immer noch nicht bekannt, dass die Hörscreeningdaten für eine Evaluation dokumentiert und in Sammelstatistiken bereitgestellt werden müssen.

Die in der Kinder-Richtlinie vorgesehenen Sammelstatistiken der Leistungserbringer dokumentieren nicht immer vollständig und plausibel die relevanten Screeningparameter. Ferner werden über Sammelstatistiken ausschließlich kumulative Daten erhoben und dies nur bis zur Entlassung aus der Klinik. Damit ist eine valide Erfassung von Qualitätsindikatoren nicht gewährleistet. Besser geeignet für die Evaluationen waren die von den HSZ für die kooperierenden Abteilungen anstelle der Sammelstatistiken erstellten anonymisierten Einzeldatensätze. Nur in diesen Einzeldatensätzen kann der gesamte Screeningprozess vom Erstscreening bis zur endgültigen Diagnose erfasst und evaluiert werden. Allerdings unterscheiden sich die Daten hinsichtlich Anzahl und Definition der erhobenen Parameter zwischen den HSZ beträchtlich. Eine Vereinheitlichung der zu erhebenden Parameter und deren Definitionen in den HSZ wurde durch den VDHZ initiiert [17] und nach den Ergebnissen der ersten Evaluation 2018 fortgesetzt (z. B. Definition Rescreening, Risikofaktoren für perinatale Hörstörungen). Eine Aufnahme dieser Definitionen in die Kinder-Richtlinie wird empfohlen.

In 13 Bundesländern arbeiten derzeit 15 HSZ, die jedoch sehr unterschiedlich organisiert und finanziert sind. Die meisten HSZ erinnern die Eltern an ein noch nicht durchgeführtes Hörscreening und notwendige Kontrolluntersuchungen nach einem auffälligen Hörtest (Tracking). So tragen sie zur Verbesserung des Screeningprozesses bei. Die Evaluationsergebnisse zur dokumentierten Screeningrate war in Bundesländern mit einer flächendeckend arbeitenden HSZ in der Regel deutlich besser. Eine positive Ausnahme war hier das Saarland ohne HSZ, in dem die vollständige Erfassung des NHS über das Modul Geburtshilfe der externen stationären Qualitätssicherung gelingt. Dieses Modell wurde 2019 auch in Baden-Württemberg etabliert. Im Gegensatz zum Saarland wurde in Baden-Württemberg zusätzlich eine HSZ für das Tracking der auffälligen Befunde eingerichtet [18] Eine Anbindung aller geburtshilflichen und neonatologischen Abteilungen an eine HSZ sollte flächendeckend für ganz Deutschland angestrebt werden, um die Qualität des NHS zu verbessern.

Der Anteil der Kinder, bei denen keine endgültige Diagnose bekannt ist, war in den HSZ sehr unterschiedlich. Unklar ist, ob bei diesen Kindern keine Kontrolle aufgrund der unterschiedlichen Intensität und Dauer des Trackings der HSZ stattfand („lost to follow-up“) oder nur die Befunde nicht durch die Nachuntersuchungsstellen übermittelt wurden („lost to documentation“). Daher sollten weitere Qualitätsanforderungen an die HSZ, wie z. B. das Tracking eines Kindes mit auffälligem Erstscreening bis zur endgültigen Abklärung und nicht nur bis zur ersten Kontrolluntersuchung oder einem bestimmten Zeitpunkt, definiert, umgesetzt und auch finanziert werden. Dies wird die Qualität des Hörscreenings in der Fläche erheblich verbessern. Wichtig wäre hierbei, dass die Ergebnisse der Kontrolluntersuchungen und der endgültigen Diagnose an die HSZ übermittelt und dort vollständig erfasst werden. Auch in anderen Ländern wird eine hohe Lost-to-follow-up-Rate von bis zu 32 % beim NHS gesehen und mögliche Verbesserungen diskutiert [19–23]. Neben der Einrichtung von koordinierenden Stellen, wie den HSZ, könnte eine Verpflichtung zur Übermittlung der Ergebnisse der Diagnostik zu einem effektiveren Tracking und einer besseren Dokumentation führen. Online-Tools erlauben eine möglichst einfache Erfassung und Übermittlung aller Daten in einer Datenbank [19].

Ein entscheidender Faktor für die Qualität und Akzeptanz eines Screening-Programmes ist die Rate an auffälligen Screeningbefunden (Refer-Rate). Auffällige Befunde müssen diagnostisch weiter abgeklärt werden und führen, auch wenn sich der Verdacht auf eine Erkrankung durch den auffälligen Befund bei der weiteren Abklärung nicht bestätigt, zu einer Verunsicherung der Eltern [24]. Eine niedrige Refer-Rate beim NHS kann vor allem durch eine gute Messqualität und einen mehrstufigen Screeningalgorithmus erreicht werden. Wichtige Faktoren für eine gute Messqualität sind fortlaufende Schulungen des Personals und einfach zu bedienende Screeninggeräte. Während der COVID-19-Pandemie wurden von einigen HSZ Online-Schulungsprogramme entwickelt, die zeitlich individuell bei Bedarf genutzt werden können. Vor Ort muss nur noch eine Einweisung in die verwendeten Screeninggeräte erfolgen [25]. Schulungen, ggf. online, sollten in den Kliniken regelmäßig durchgeführt werden, vor allem bei häufigem Personalwechsel.

Ein Rescreening nach auffälligem Erstscreening führte zu einer deutlichen Senkung der Refer-Rate, da diese zweite Untersuchung in 81,3 % (81,6 %) der Fälle einen unauffälligen Befund ergab (Tab. 1). Insgesamt wurde nach einer auffälligen Messung bei ca. 75,0 % der Kinder eine weitere Messung dokumentiert. Diese konnte allerdings nur bei ungefähr der Hälfte der auffälligen Befunde im Sinne eines Rescreenings berücksichtigt werden. Problematisch ist, dass häufig zwischen einer erneuten Testung im Rahmen des Erstscreenings oder einem Rescreening nicht differenziert werden kann. Auch werden in einigen Kliniken teils sehr viele Messversuche durchgeführt, um das Neugeborene mit einem unauffälligen Hörtest entlassen zu können, so dass es statistisch alleine durch sehr häufige Wiederholungen rein zufällig zu einem unauffälligen Befund des NHS kommen könnte [26]. Als Gründe für nicht durchgeführte Rescreenings wurden in den Interviews ambulante Entbindungen, immer frühere Entlassungszeitpunkte und Personalmangel genannt. Darüber hinaus werden in den Sammelstatistiken Rescreenings nicht erfasst, wenn die Eltern für diese Untersuchung in kooperierende Abteilungen in der Klinik einbestellt werden.

Die als Rescreening dokumentierten Messungen wurden in über der Hälfte der Fälle, entgegen den Vorgaben der Richtlinie, mit einer TEOAE durchgeführt. Begründet wurde dies in den Interviews mit einer deutlich längeren Messdauer und erhöhter Störanfälligkeit der AABR. Das macht gerade bei Personalmangel die TEOAE-Messungen attraktiv. In den Jahren 2017/2018 bestand auch immer noch für ca. 16 % der Geburtsabteilungen keine Möglichkeit, eine AABR-Messung selbst oder in Kooperation mit einer anderen Abteilung durchzuführen. In der Folge-Evaluation konnte gezeigt werden, dass die Rate der auffälligen Ergebnisse des Rescreenings bei einem Methodenwechsel deutlich höher ist und bei dem Algorithmus TEOAE/TEOAE mit 9,6 % die niedrigste Rate auffälliger Rescreening-Befunde erreicht werden kann. Vor diesem Hintergrund sollte nach einem auffälligen Erstscreening mit TEOAE auch ein Rescreening mit TEOAE akzeptiert werden. Damit kann die Anzahl der durchgeführten Rescreenings erhöht und die Refer-Rate verbessert werden.

Dieses Vorgehen entspricht den aktuellen internationalen Empfehlungen. Das Joint Committee on Infant Hearing empfiehlt in seinem Positionspapier 2019 mindestens zwei Screeningversuche mit der gleichen Methode oder eine AABR-Kontrolle nach TEOAE vor Entlassung eines Kindes ohne Risikofaktoren („well babies“). TEOAE-Kontrollen nach einer auffälligen AABR werden inzwischen bei „well babies“ akzeptiert, da bei ambulant durchgeführten notwendigen Kontrollen die Lost-to-follow-up-Rate sehr hoch ist [23, 26]. In den Vorgaben des englischen Screeningprogramms ist nach einem auffälligen TEOAE bei „well babies“ zunächst ein weiterer TEOAE-Test mit einem zeitlichen Abstand von mindestens 5 h vorgesehen und die Zahl der Testversuche wird auf 3 begrenzt [27]. Die Compliance des screenenden Personals für die Durchführung eines Rescreenings könnte durch einen Screeningalgorithmus mit Begrenzung der Testversuche und der Möglichkeit eines TEOAE-Rescreenings verbessert werden. Bei Kindern mit Risikofaktoren für perinatale Hörstörungen sollten Erst- und Rescreening immer mit einer AABR durchgeführt werden [8, 26, 27].

In der ersten Evaluation wurde die weitere Abklärung nach einem auffälligen Befund aus den Daten der HSZ evaluiert. Es zeigte sich, dass eine erste Kontrolluntersuchung nach einem auffälligen Befund bei Entlassung entgegen der Richtlinie häufig in einer HNO- oder pädiatrischen Praxis und nicht in einer pädaudiologisch qualifizierten Institution durchgeführt wurde. Diese erste Kontrolle war 2012 bei 82,9 % der Kinder unauffällig. Die Lost-to-follow-up-Rate nach einem auffälligen Screeningbefund kann durch Zulassung (und Vergütung) eines niederschwelligen wohnortnahen ersten Kontrollscreenings in einer Praxis deutlich reduziert werden, da dieses von den Eltern einfacher wahrgenommen werden kann. Allerdings muss diese Untersuchung auch mit objektiven Methoden wie TEOAE oder AABR und nicht nur klinischen Tests oder einer Tympanometrie durchgeführt werden [28]. Dieses Vorgehen entlastet auch die pädaudiologischen Einrichtungen. Zeigt diese erste Kontrolluntersuchung jedoch erneut ein auffälliges Ergebnis, müssen diese Kinder zeitnah in einer pädaudiologisch qualifizierten Institution vorgestellt werden. Weitere Kontrollen in einer HNO-Praxis sind nicht zielführend und verzögern die Diagnosestellung.

In der ersten Evaluation wurde die Prävalenz einer beidseitigen permanenten Hörstörungen aus den pädaudiologischen Daten der Bundesländer mit vermutlich nahezu vollständiger Erfassung von 1,3 auf 1000 Neugeborene berechnet. Dies entspricht der aus der Literatur erwarteten Größenordnung [29].

Zur weiteren Optimierung des NHS in Deutschland wurden in beiden Evaluationsberichten Empfehlungen und Vorschläge gemacht (s. Infobox).

## Fazit

Beim Vergleich der Ergebnisse der Folge-Evaluation mit denen der ersten Evaluation zeigte sich, dass das NHS insgesamt besser dokumentiert war und mehr Kliniken an eine HSZ angebunden sind. Die Qualität des Hörscreenings hatte sich nicht verbessert. Die Refer-Rate hat sich verschlechtert und die in der Richtlinie definierten Zielkriterien sind noch nicht flächendeckend erreicht. Auch wenn in einigen Bereichen noch Herausforderungen bestehen, zeigen die vom G‑BA in Auftrag gegebenen Evaluationen für die Jahre 2011/2012 und 2017/2018 für Deutschland ein insgesamt erfolgreich umgesetztes Hörscreening [11, 12]. Bei über 85 % der in Deutschland geborenen Kinder wurde 2018 ein Hörscreening durchgeführt. Ein großer Erfolg ist die deutliche Vorverlegung des Diagnose- und Therapiezeitpunkts. Wurde die Diagnose einer beidseitigen Hörstörung vor Einführung eines flächendeckenden Screenings auch bei einer mittelgradigen Hörstörung im Mittel erst im Alter von über 4 Jahren gestellt [30], so lag 2012 der Median des Diagnosealters bei 4 Monaten und der Therapiebeginn bei mehr als der Hälfte der Kinder vor dem 6. Lebensmonat.

### Infobox Optimierung des NHS in Deutschland – Beispiele für Empfehlungen und Vorschläge aus den beiden Evaluationsberichten

- Aufnahme eindeutiger Definitionen in die Kinder-Richtlinie (z. B. Abgrenzung Erst- und Rescreening, Risikofaktoren)
- Flächendeckende Einrichtung von Hörscreening-Zentralen (HSZ) mit verpflichtender Anbindung aller Leistungserbringer an eine HSZ
- Vorgabe von Qualitätskriterien an die HSZ, wie z. B. Tracking und einheitliche Erfassung der Daten bis zur endgültigen Diagnose; hierfür wäre eine entsprechende personelle Ausstattung erforderlich, die auch finanziert werden müsste
- Verbesserung der Refer-Rate durch ein konsequentes Rescreening mit definiertem Algorithmus, bei Kindern ohne Risikofaktoren für perinatale Hörstörungen auch mit einer weiteren TEOAE als Screeningmethode
